# Development and Application of Loop-Mediated Isothermal Amplification (LAMP) Assays for Rapid Diagnosis of the Bat White-Nose Disease Fungus *Pseudogymnoascus destructans*

**DOI:** 10.1007/s11046-022-00650-9

**Published:** 2022-08-05

**Authors:** Ludwig Niessen, Marcus Fritze, Gudrun Wibbelt, Sebastien J. Puechmaille

**Affiliations:** 1 TUM School of Life Sciences, Technical University of Munich, Gregor-Mendel-Str. 4, 85354 Freising, Germany; 2 Applied Zoology and Nature Conservation, University of Greifswald, Loitzer Str. 26, 17489 Greifswald, Germany; 3 German Bat Observatory, Am Juliusturm 64, 13599 Berlin, Germany; 4 Leibniz Institute for Zoo and Wildlife Research, Alfred-Kowalke-Straße 17, 10315 Berlin, Germany; 5 ISEM, CNRS, EPHE, IRD, University of Montpellier, Montpellier, France; 6 Institut Universitaire de France, 75005 Paris, France

**Keywords:** White-Nose Disease, Pseudogymnoascus destructans, Bat, Molecular detection, Neutral red indicator, Tape lifting, Swabbing, Field trial

## Abstract

*Pseudogymnoascus destructans* (= *Geomyces destructans*) is a psychrophilic filamentous fungus that causes White-Nose Disease (WND; the disease associated with White-Nose Syndrome, WNS) in hibernating bats. The disease has caused considerable reductions in bat populations in the USA and Canada since 2006. Identification and detection of the pathogen in pure cultures and environmental samples is routinely based on qPCR or PCR after DNA isolation and purification. Rapid and specific direct detection of the fungus in the field would strongly improve prompt surveillance, and support control measures. Based on the genes coding for ATP citrate lyase1 (*acl1*) and the 28S-18S ribosomal RNA intergenic spacer (*IGS*) in *P. destructans*, two independent LAMP assays were developed for the rapid and sensitive diagnosis of the fungus. Both assays could discriminate *P. destructans* from 159 tested species of filamentous fungi and yeasts. Sensitivity of the assays was 2.1 picogram per reaction (pg/rxn) and 21 femtogram per reaction (fg/rxn) for the *acl1* and *IGS* based assays, respectively. Moreover, both assays also work with spores and mycelia of *P. destructans* that are directly added to the master mix without prior DNA extraction. For field-diagnostics, we developed and tested a field-applicable version of the *IGS*-based LAMP assay. Lastly, we also developed a protocol for preparation of fungal spores and mycelia from swabs and tape liftings of contaminated surfaces or infected bats. This protocol in combination with the highly sensitive *IGS*-based LAMP-assay enabled sensitive detection of *P. destructans* from various sources.

**Supplementary Information:**

The online version contains supplementary material available at 10.1007/s11046-022-00650-9.

## Introduction

During February 2006, the first evidence of North-American hibernating bats displaying visible signs associated with white-nose disease (WND) was documented in a cave near Albany, NY [1]. During subsequent years, the disease spread from New York to adjacent U.S. states and is now found in 38 U.S. states and seven Canadian provinces (see https://www.whitenosesyndrome.org/ for a current map) and recent studies showed that several bat species in North America show a population level response with a significant decrease in presence and abundance post-WND [2–4]. The causative agent of WND has been identified as the psychrophilic fungus *Pseudogymnoascus destructans* Minnis & D.L. Lindner (= *Geomyces destructans* Blehert & Gargas) [1, 5, 6]. Aside from North America, the fungus is prevalent in the Western Palearctic and Palearctic Asia [7–12]. However, in the Palearctic, observed mortality in bat colonies with confirmed *P. destructans* presence was similar to colonies from which *P.* *destructans* was not reported [11, 13]. The absence of mass mortality in the Palearctic combined with phylogenetic studies of European, North American and Asian *P. destructans* populations suggest that the fungus was introduced to North America from Europe rather than from Asia, even though the definitive source of the invading isolate has not yet been conclusively identified [14, 15].

For diagnostics, the detection and identification of *P. destructans* and its distinction from closely related species largely relies on molecular techniques such as quantitative PCR (qPCR). Single- or multi locus sequencing [6, 16] or microsatellite analysis [17–21] have also been used in research studies. Molecular techniques use genomic DNA that has to be isolated and purified from pure culture mycelia or sample materials before the analysis [22, 23]. However, due to its slow growth (8 mm in 16 d at 14 °C), cultivation of *P. destructans* is time consuming [5]. To aid diagnosis of the fungus, diagnostic PCR and qPCR assays were established for several genes: the 18S region of the rRNA gene [24, 25], the gene coding for alpha-L-rhamnosidase (*ALR*) [26], the rRNA intergenic spacer (*IGS*) region [27] and the rRNA internal transcribed spacer (ITS) region [28]. However, DNA also needs to be extracted and purified before running these assays.

Loop-mediated isothermal amplification (LAMP) [29] is an alternative technology for DNA-based diagnosis of microorganisms that runs under isothermal conditions at 65 °C. The specificity of the LAMP reaction is higher compared to qPCR because a set of four primers with six binding sites must hybridize correctly to their target sequence before autocycling DNA biosynthesis occurs. Amplification events can be detected visually by in-tube colour reaction of indicator dyes such as calcein in a fluorescence based assay [30] or neutral red in systems used under day light conditions [31]. Over the past 15 years, LAMP-based assays were established for a number of yeast-like and filamentous fungal species. Assays can be used to identify pure fungal cultures or to detect the target organisms in other sample types [32, 33]. Together with its lower sensitivity to typical polymerase inhibitors [34], the LAMP technology provides a versatile technology for rapid, user-friendly and inexpensive diagnostic assays that can be used in simple lab settings or even under field conditions. Such features make LAMP assays an ideal tool for the study of *P. destructans*.

We report the development and application of LAMP based assays for the rapid identification of *P. destructans* in pure cultures and other sample types as well as the development of innovative, rapid and simple protocols for the preparation of fungal material for testing with LAMP-based methods in the field.

## Materials and Methods

### Organisms and Culture Conditions

A total of 217 fungal cultures have been used during the current study (complete list in Table S1 of supplemental materials). The cultures originated from two sources. The large majority of non-*Pseudogymnoascus* sp. cultures were obtained from various official culture repositories or from collections of fungal cultures maintained at public research institutions (see column 3 and footnotes in Table S1, strains marked in green) while the majority of *Pseudogymnoascus* sp. cultures came from the authors reference collection (see further details in Table S1).The 53 cultures of *P. destructans* that were used in the current study were isolated from bat swabs, bat guano or bat hibernacula environments (sediments/walls) from various locations in Europe and North America [8, 15, 20, 35]. The self-isolated fungal strains shown in table S1 (marked in grey) were identified to species/clades level using micromorphology, FTIR spectroscopy or genetic methods (LAMP assay [36, 37], gene sequencing, e.g. 28S rRNA, ITS, beta tubulin, calmodulin, or multi locus sequencing [15]). Part of the pure cultures of *P. destructans* were identified using microsatellite analysis as described in [18] based on published markers from [17]. For analysis of fungi in bat guano from nine German roost sites (6 in Hesse and 3 in Baden Wuerttemberg, 2010 and 2011) as well as two samples of soil from Czech bat caves, bat guano pellets and soil particles were placed on Sabouraud´s dextrose agar (SDA, 0.5% (w/v) peptone from casein, 0.5% (w/v) meat peptone, 2% dextrose, 1.2% (w/v) agar) and incubated at 13 °C in the dark. Colonies were transferred to fresh SDA plates for pure culturing. For identification of strains, colonies that resembled *Pseudogymnoascus* spp. were maintained on SDA. Colonies of other species were re-inoculated to malt extract agar (MEA, 3% w/v malt extract (ApliChem, Darmstadt, Germany), 0.3% w/v soy peptone [Karl Roth, Karlsruhe, Germany), 2% w/v agar, pH 5.8) prior to microscopic identification using micromorphological characteristics and appropriate identification keys [38–40]. Cultures of environmental fungi that could not be readily identified microscopically were identified according to signature sequences, e.g. beta tubulin gene for *Penicillium* spp., calmodulin gene for *Aspergillus* spp., transcription elongation factor 1 alpha for *Fusarium* spp. and rRNA internal transcribed spacer (ITS) for all other genera.

For long-term storage, cultures were maintained as cryo-cultures at -80 °C in 80% glycerol as described previously [41]. Working cultures of non-*P. destructans* isolates were obtained from cryo-cultures by inoculation to MEA and incubation at ambient temperature under diffuse day light. Isolates of *P. destructans* and closely related taxa were cultivated on SDA at 13 °C in the dark. All *Fusarium* spp. were grown on Synthetic Nutrient Agar (SNA, [42]) amended with 0.4 g of glucose at ambient temperature under diffuse day light.

Twenty-one field samples were collected from bats (n = 10; 8 swabs and 2 tape liftings) and bat hibernacula walls (n = 11 swabs) towards the end of the hibernation season in March/April [20]. Sampled bats were found either with (n = 7) or without (n = 3) visual fungal growth indicating *P. destructans* infection [66]. Swabs were stored in 1.5 mL tubes and tape liftings were stored on microscope slides, at ~ 5 °C until usage.

### DNA Preparation from Pure Cultures

PCR grade genomic DNA was prepared from 30 mL liquid cultures of the 217 isolates grown under conditions that were optimum for the respective species. Mycelia were vacuum filtered and washed twice with sterile tap water. Fifty milligram of mycelia were treated for 1 min in a Fastprep® 24 system with addition of sterile glass beads (0.4 g of 0.5 mm diameter and 0.22 g of 1.25–1.65 mm diameter) at an amplitude of 6 m/s in lysis buffer PL2 provided with the peqGold Fungal DNA Mini Kit (VWR International, Erlangen, Germany). DNA purification from treated mycelia was performed according to the manufacturer´s protocol. DNA was maintained frozen at − 20 °C until used and further stored at 4 °C after initial thawing.

### DNA Sequencing

A 766 bp partial sequence of the multicopy 28S-18S ribosomal RNA intergenic spacer (*IGS*) of *P. destructans* isolate 01NH07 (GenBank accession number JX270192; [43]) was downloaded from GenBank for primer design. To design an alternative primer set, from a single copy gene, the coding sequence of the *P. destructans* ATP citrate lyase subunit 1 gene (*acl1*) was retrieved from GenBank (isolate 20631-21, GenBank accession number XM_012884837). Since the *P. destructans* type 20631-21 was unavailable to our study, we used isolate *P. destructans* OT-38-2010 (isolated from *Myotis mystacinus*, Finnentrop, Germany; culture ‘Mmys-DE-2’ in [11]) as reference throughout the study*.* Presence and correctness of the *acl1* sequence in the reference isolate *P. destructans* OT-38-2010 was checked by sequencing three overlapping portions of the gene using primers Geo-*acl1*-f to Geo-*acl1*-r2 (see Table 1 for primer sequences. See method descriptions in supplemental materials for a detailed description of *acl1* sequencing).

**Table 1:** List of primers used during the current study

Primer name	Sequence 5′ > 3′	PCR annealing (°C)
*Sequencing primers for acl1 gene*		
Geo-acl1-f	CAC AAC CGT ACC GTC AAT TG	57
Geo-acl1-f1	CTT TGA GCC CTC GTG CTG AG	61.5
Geo-acl1-f2	ATC CCT CTC GAT TAC TCG TG	59
Geo-acl1-r	TAG CAT AGC GCG TCA AAC TC	57
Geo-acl1-r1	AGC TCC ATG GTG GAC TGG TAC A	61.5
Geo-acl1-r2	TAC AGA AGC TCC TGG CCA CG	59
*Sequencing primers for LAMP products*		
F2-Pd-acl1	CGC GAG CAT GTT CAA GAC C	58
B2-Pd-acl1	GG GCA TGT CCT CGA AGG T	58
F2-Pd-IGS	C TGG CGT TAC AGC TTG CT	54
B2-Pd-IGS	T CGA AAC TGG ACC CAT TTG G	54
*acl1-specific LAMP primers*		
^b^F3-Pd-acl1-ID30	ATT GTT GCC TGG GCG ATC	
B3-Pd-acl1-ID30	TAC ACA GAG CTG AGG AGT GC	
^c^FIP-Pd-acl1-ID30	^a^CTG CGA GTT GGC GAA CGA TCC-CGC GAG CAT GTT CAA GAC C	
^c^BIP-Pd-acl1-ID30	^a^GAC CGC TGC CAA CAA GAA CAA G-GG GCA TGT CCT CGA AGG T	
^d^LF-Pd-acl1-ID30	AGC GTG TCC GAA CTG AAC CT	
^d^LB-Pd-acl1-ID30	TGA AGG AGG CCG GCT TCC A	
*IGS-specific LAMP primers*		
^e^F3-Pd-IGS-ID10	CCT CTC CGC CAT TAG TGC	
^e^B3-Pd-IGS-ID10	GCT CCG ACG TAA TAG GTG C	
FIP-Pd-IGS-ID10	AGG TAG GTG AGC TAC CCG GC-C TGG CGT TAC AGC TTG CT*	
BIP-Pd-IGS-ID10	GAA GTC GCA GAG TGG CCC TG-T CGA AAC TGG ACC CAT TTG G*	
LF2-Pd-IGS LF1-Pd-IGS-ID36	AAA GCA GCC ACC ACC GGC T AAC CAG CTA GAG AGG CAG C (only used in experiments with V13-01184 dye)	
LB-Pd-IGS-ID36	CGC GTC CCT TTT TAC AAA ATG C	

^a^Hyphen indicates junction between F1c/B1c (left) and F2/B2 (right) parts of FIB/BIP primers

^b^See Notomi et al. (2000) for an explanation of the positioning of LAMP primers on the target DNA

^c^FIP = forward inner primer; BIP = backward inner primer

^d^LF = loop forward primer; LB = loop backward primer

^e^F3 = forward outer primer; B3 = backward outer primer

Identity of LAMP products was verified by sequencing of the smallest amplified fragments. LAMP reactions were performed with primer sets *IGS*-ID10 and *acl1*-ID30, respectively and separated on 1.5% agarose gels. The smallest DNA fragment was cut from each of the two gels and purified using the QIAquick Gel Extraction Kit (QIAGEN, Hilden, Germany) according to the manufacturer's recommendations. Each of the purified LAMP fragments was re-amplified by PCR using primers F2-Pd-*acl1*/B2-Pd-*acl1* and F2-Pd-*IGS*/B2-Pd-*IGS*, respectively (see Table 1) according to the following cycling protocol: 1 cycle 5 min initial melting at 95 °C, 40 cycles consisting of 1 min initial melting at 95 °C, 1 min annealing at 54 °C (primers Pd-*IGS*) or 58 °C (primers Pd-*acl1*), 1 min elongation at 72 °C, and 1 cycle of 5 min final elongation at 72 °C. Amplification products were purified using the QIAquick PCR Purification Kit (QIAGEN, Hilden, Germany) and sent off for bi-directional Sanger sequencing using PCR primers as sequencing primers. Consensus sequences were created and aligned using the BioEdit Software package [44]. The resulting consensus sequence was aligned to the *acl1* and *IGS* gene sequences of *P. destructans* 20631-21, respectively. For verification, BLASTn [45] was used to search the NCBI nucleotide database for homologies with the consensus sequences from each of the two target genes (*IGS* and *acl1*).

Gene copy numbers were calculated from gDNA concentrations using the calculator available at the home page of the University of Rhode Island CELS Genomics and Sequencing Center (http://cels.uri.edu/gsc/cndna.html).

### LAMP Primer Design

Primers for the LAMP *acl1*-specific assay were designed manually while those for the *IGS*-specific assay were designed using the PrimerExplorer software available on the Eiken GENOME website (see http://primerexplorer.jp/e/) (see Table 1 for primer sequences). Primer sets were selected to have a dimer(minimum)dG-value close to − 2.0, respectively. Default settings were applied for all other parameters adjustable in the primer design software. In silico analysis of primer specificity was done by testing each primer in a BLASTn [45] search against the NCBI sequence database. Target gene sequences of several *Pseudogymnoascus* spp. and from other fungal species for which > 90% similarities were found in at least three of the tested primers were extracted from the respective genome assemblies. Each primer was aligned with a multiple alignment (CustallW, BioEdit v7.2.5 [44]) of the respective target gene in the selected species for specificity analysis.

### Vacuum Drying of LAMP Master Mix (‘dry-LAMP’)

A master mix consisting, per reaction, of 8 mM MgCl_2_, 1.4 mM each deoxynucleotide (dG, dA, dT, dC), 1.6 µM primers FIP/BIP, 0.8 µM primers LF/LB, 0.2 µM primers F3/B3, 0.1 mM neutral red, 8 U *Bst* 2.0 Warmstart DNA-polymerase (New England Biolabs, Frankfurt/M., Germany) and 15.9 µL sterile demineralized water was prepared and 25 µL per reaction were distributed into 200 µL PCR tubes (8-Strip PCR Tube, individually attached flat cap, optically clear, Star Lab, Hamburg, Germany). The master mixes were vacuum dried in a desiccator with the tube lids opened at ambient temperature (22 °C) and 3 mbar pressure for 60 min. For quality control, two LAMP reactions were tested directly after drying by adding 25 µL of *P. destructans* OT-38-2010 gDNA (2.4 ng/µL) in 1 × amplification buffer (10 mM (NH_4_)_2_SO_4_, 10 mM KCI, pH 8.7) as positive control and 25 µL 1 × amplification buffer without gDNA as negative control. Dried LAMP reactions (‘dry-LAMP’) were sealed in clear plastic bags together with a silica desiccant pack and stored at 4 °C in the dark until use.

### Specificity Testing of LAMP Assays

LAMP assays were run under the conditions shown in Table 2 using calcein as fluorescence indicator and purified genomic DNA of the fungal isolates listed in Table S1 as template. Five microliters of DNA at 20 ng/µL was added to the LAMP reactions to ensure that even in case of a weak cross-reaction, a LAMP signal would occur. Purified genomic DNA of 217 fungal isolates representing 159 different species, subspecies and varieties from 33 genera of filamentous fungi and yeasts was tested for cross reactivity with both LAMP assays. Among others, DNA of 53 isolates of *P. destructans* originating from 12 countries were tested as reference material.

**Table 2:** Composition of master mixes (25 µl per reaction) and amplification conditions used during the current study

Component	LAMP Pd-acl1-ID30 Calcein	LAMP Pd-IGS-ID10 Calcein	LAMP Pd-IGS-ID10 Neutral red	LAMP Pd-IGS-ID10 V13-01184
10 × LAMP buffer	2.5	2.5	2.5	2.5
MgCl_2_ (250 mM)	1.0	1.0	1.0	1.0
dNTP’s (10 mM each A,T,G,C)	3.5	3.5	3.5	3.5
100 × primer mix	2.6	2.6	2.6	2.6
ultrapure water	7.8	7.9	7.45	7.65
formamide (100%)	0.6	0.5	0.95	1.0
Bst polymerase (8 U/µl)	1.0	1.0	1.0	1.0
Calcein reagent (1.25 mM)	1.0	1.0	n.a.*	n.a
Neutral red (2.5 mM)	n.a	n.a	1.0	n.a
V13-01184 dye (578.48 g/mol)	n.a	n.a	n.a	1.0
DNA sample	5.0	5.0	5.0	5.0
Total volume	25.0	25.0	25.0	25.0
*Conditions*				
Incubation temperature (°C)	64.0	65.0	65.0	65.0
Incubation time (min)	60	60	60	60

^*^n.a. = not applied

### Preparation of Tape Lifting Samples

The tape lifting technique was developed based on a previously described touch imprint method used to obtain samples from *P. destructans* infected bats for microscopic and PCR analysis [7]. We used two types of adhesive tape, a common household/office tape (tesafilm®, BNR 57284, tesa SE, Norderstedt, Germany), similar to the tape that has been used so far [7] and Water Soluble Wave Solder tape (Scotch™ Brand Mask Plus II 1″ × 36 yrd no. 5414, 3 M Industrial Tape Division, St. Paul, MN, USA). For both materials, strips of approximately 4 cm were cut from a roll and pressed to a sampling surface with its adhesive side. After removal from the sampling surface, the tape was either adhered to a microscope glass slide or folded with the adhesive sides of both ends touching and transferred to a sterile 2.0 mL round bottom reaction vessel after two more foldings (only for the water soluble tape).

For the tesafilm® tape lifting samples (field experiment), small pieces (~ 1 to 2 cm^2^) containing lifted sample material were directly transferred into 300 µL LAMP buffer and 25 µL of this template-buffer-solution was used in the dry-LAMP reaction (see field experiment section).

Since tesafilm® can very easily stick to the tube and thus LAMP buffer might not always reach the tape areas with spores, we developed an alternative method using water soluble tape. The water soluble tape was solubilized by addition of 2 mL sterile 8 M aqueous urea (for details see method descriptions in supplemental materials). Five microliters of the resulting solution were used as template in the LAMP reaction.

### LAMP-Based Analysis of Pure Culture Material

We evaluated the potential of the *IGS*-based LAMP assay to detect *P. destructans* after direct addition of fungal material into the master mix. Minute amounts of *P. destructans* pure culture material including mycelium and spores were removed from SDA grown colonies with either a heat sterilized steel needle or a sterile swab from which spores were later obtained by centrifugation in demineralized water. Cell material (dry or in solution) was transferred directly to the LAMP master mix prior to incubation at 65 °C for 60 min. For testing the influence of culture age on direct detection of fungal material, samples were collected from colonies daily, starting at day three until day 31 of cultivation at 13 °C on SDA. Since colonies started developing guttation droplets from day 23 onward, these colonies were sampled both from the margin and centre and samples were tested in separate LAMP reactions.

### Isothermal DNA Amplification

Loop-mediated isothermal amplification (LAMP) of *P. destructans* DNA was performed using essentially the protocol described by Tomita, Mori [30] with slight modifications. The master mix was prepared containing per 25 µL reaction: 2.5 µl 10 × LAMP buffer (200 mM 3-(N-morpholino)propanesulfonic acid (MOPS), 100 mM KCl, 100 mM (NH_4_)_2_SO_4_, 80 mM MgCl_2_, pH 8.8, all chemicals from Sigma-Aldrich (Taufkirchen, Germany) and Carl Roth (Karlsruhe,Germany), 3.5 µL dNTP mix (10 mM each, MP Biomedicals, Heidelberg, Germany), 2.6 µL primer mix (resulting in final concentrations of 1.6 µM each FIP and BIP, 0.8 µM each LF and LB, 0.2 µM each F3 and B3 in the master mix, see Table 1 for sequences, all primers by Eurofins, Ebersberg, Germany), 8 U (1.0 µl) *Bst* DNA polymerase, large fragment (8000 U/mL, New England Biolabs, Frankfurt, Germany), 1 µL calcein reagent (see Niessen and Vogel [46]), or V13-01184 dye (10 µg/mL working solution (Dyomics, Jena, Germany] [47], or neutral red reagent [31, 48]. Demineralized water was added to result in a 25 µL total reaction volume, including sample DNA. Regularly, 5 µL of template DNA solution or spore suspension were added per reaction. Visual detection under day light conditions was achieved using neutral red as indicator dye. In this case the 10 × LAMP buffer was composed of 100 mM (NH_4_)_2_SO_4_, 100 mM KCI, pH 8.7. Reaction components used for the different LAMP assays are listed in Table 2. Twenty microliters of the master mix per sample were distributed into 200 µL PCR tubes (8-Strip PCR Tube, individually attached flat cap, optically clear, Star Lab, Hamburg, Germany) and 5 µL sample DNA was added. DNA of *P.* *destructans* OT-38-2010 (1.0 ng/reaction) was used as positive control in both assays throughout the study. Water was added instead of DNA in no template controls (NTC). Sterile filter pipette tips were used for all liquid handling (Star Lab, Hamburg, Germany). Two different sets of pipettes were used for DNA-free operations and operations involving DNA, respectively. Preparation of master mixes and addition of sample DNA were performed in separate rooms. Agarose gel electrophoresis of LAMP products was performed in a room separate from master mix preparation and template addition. Reaction tubes were placed in the heating block of a Mastercycler® gradient thermal cycler (Eppendorf, Hamburg, Germany) operated at constant temperature according to the optimized protocol for respective LAMP reactions (Table 2). For inspection of calcein-based assays, reaction tubes were placed upon black photo cardboard under a 365 nm UV lamp (MinUVIS, Desaga, Heidelberg, Germany) and inspected for the presence of a bright green fluorescence. Neutral-red based assays were documented by placing tubes on a white-light transilluminator. Results were documented using a hand held digital camera.

### Detection of *P. destructans* in Field Swab and Tape Samples Using dry-LAMP

We used dry-LAMP reactions to test two different field sample types: (1) nylon swabs (CLASSIQSwabs, COPAN, Brescia, Italy) collected from bats and hibernacula walls and (2) tesafilm® (tape liftings) collected from bats. One negative control was included every 7th sample. Swabs and pieces of the tesafilm® (~ 1 to 2 cm^2^) were transferred directly to 300 µL LAMP buffer in 1.5 mL tubes and vigorously shaken by hand for 1 min and 25 µL of the solution was transferred to the dry-LAMP tubes. After brief manual shaking until the dry-LAMP pellet was dissolved, tubes were incubated at 65 °C for 60 min using a Heating Thermomixer (HLC BioTech, Bovenden, Germany). All dry-LAMP procedures and reactions were run outside a laboratory on an office desk (to mimic field conditions). Surfaces and materials (pipettes, scalpel blades, forceps) were cleaned and disinfected with 70% aqueous 2-propanol solution.

## Results

### Sequence Determination of the *acl1* Gene in *P. destructans*

BLASTn [45] was used to search the NCBI nucleotide database for the *acl1* gene in the genomic sequence of *P. destructans* strain 20631-21. The search retrieved a 2067 nt sequence between positions 1,116,082 and 1,118,148 on the unplaced genomic scaffold_9 of the *P. destructans* whole genome shotgun sequence (locus_tag = VC83_04411) [49]. The coding sequence included a start and stop codon with one intron of 57 nt between nucleotide positions 481 and 537. The translated protein sequence (Expasy translate tool http://web.expasy.org/translate/) consisted of 667 amino acids. SignalP 3.0 Server analysis [50] predicted no signal peptide providing evidence that the protein is not secreted. Sequencing of the *acl1* gene of the reference isolate OT-38-2010 (GenBank Accession Number JN242242) confirmed the presence of the *acl1* gene. Both sequences showed 99.9% homology. Two nucleotide exchanges were found at positions 527 (A–T) and 540 (T–C), i.e. in the intron sequence of the gene and will therefore not alter the amino acid sequence of the resulting protein in comparison to the type strain.

### Primer Design and LAMP Product Verification

**Fig. 1:**
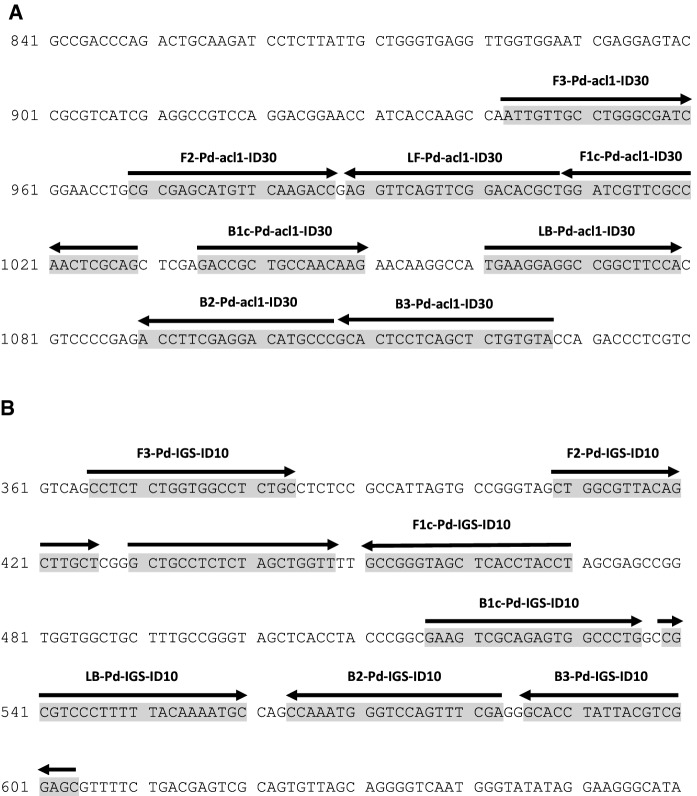
Localization of LAMP primers within their target sequences. Binding sites of LAMP primer sets for specific detection of *P. destructans* are marked in grey. **A** Partial sequence of the *acl1* gene (nucleotide positions 841-1130 of 2067, Genbank accession number JN242242) in *P. destructans* with the smallest LAMP fragment predicted to be 139 nt. **B** Partial sequence of the 18S-28S ribosomal RNA intergenic spacer *IGS* (nucleotide positions 361-659 of 766, partial sequence of Genbank accession number GQ489025) in *P. destructans* with the smallest LAMP fragment predicted to be 175 nt → indicates forward primers, ← indicates backward primers

Two sets of LAMP primers, including a pair of loop primers for each gene, were designed on the basis of the *acl1* gene and *IGS* sequences (Table 1 and Fig. 1). Similarity analysis of *acl1* and *IGS* sequences for each primer-binding site in *P. destructans* as well as other *Pseudogymnoascus* spp. and other fungal species that showed high in silico sequence similarities for some of the primers is shown in Figs. S1 and S2, respectively. The binding sites of all Pd-*acl1*-ID30 primers were in 100% homology with the corresponding sequences of two *P. destructans* strains for which *acl1* sequences were available (see Fig. S1). Moreover, the binding sites of three out of eight Pd-*acl1*-ID30 primers shared 100% identity with the respective homologues sequences of eight *Pseudogymnoascus* spp. of yet unidentified taxonomic association to *P. destructans*. Moreover, four out of eight primers showed only 1–2 mismatches. Only primer B3-Pd-*acl1*-ID30 shared a higher degree of sequence dissimilarity with the analysed *Pseudogymnoascus* spp. isolates. Non-*Pseudogymnoascus acl1* sequences showed between 0 and 10 mismatches with the Pd-*acl1*-ID30 primer sequences. No *acl1* sequences were available for *P. pannorum* and *P. verrucosus*. Figure S2 shows that all *IGS*-specific primers shared 100% homology with the respective binding sequences in two *P. destructans* strains but showed a high percentage of mismatches with 10 *Pseudogymnoascus* spp. isolates as well as *P. verrucosus*. No *IGS* sequence was available from the genome of *P. pannorum*.

**Fig. 2:**
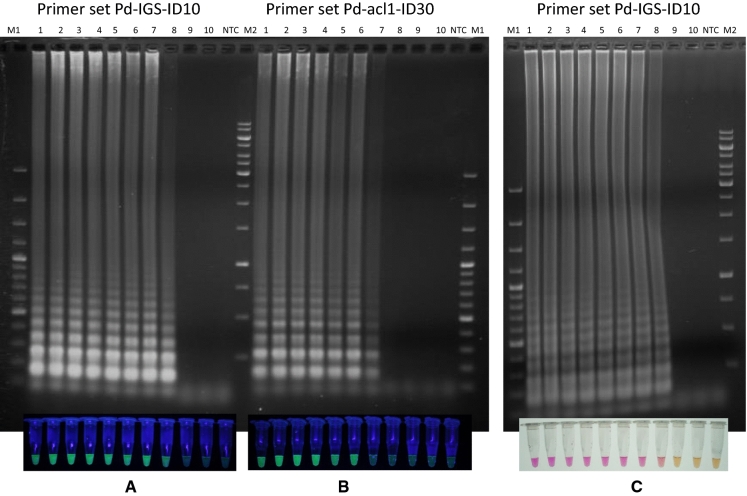
Confirmation of DNA biosynthesis in LAMP reactions and correlation with calcein fluorescence and neutral red colour change. The LAMP reactions shown in Figure S3 (A, B, C) were separated on 1.5% agarose gels and stained with dimidium bromide. LAMP reactions were electronically mounted onto respective agarose gels for comparison between the presence of DNA and calcein fluorescence/neutral red colour change. M1 = Gene ruler 100 bp DNA ladder; 1 = 213 ng per reaction (ng/rxn) of *P. destructans* OT-38–2010 gDNA; 2 = 21.3 ng/rxn; 3 = 2.1 ng/rxn; 4 = 210 pg/rxn; 5 = 21 pg/rxn; 6 = 2.1 pg/rxn; 7 = 210 fg/rxn; 8 = 21 fg/rxn; 9 = 2.1 fg/rxn; 10 = 210 ag/rxn; NTC = not template control, demineralized water instead of DNA-template figureM2 = Gene ruler 1 kb DNA ladder

Presence of a ladder-like banding pattern that typically occurs in LAMP reactions was verified by agarose gel electrophoresis (AGE) of reactions run with a serial dilution of template DNA from *P. destructans* OT-38-2010. The ladder-like pattern of DNA fragments of sizes between 150 bp and several Kbp occurred with primers specific for the *acl1* and *IGS* sequence, respectively (Fig. 2). Comparison of banding patterns and LAMP reaction tubes made it obvious that DNA was synthesized in all reactions where the calcein indicator showed a bright green fluorescence (Fig. 2A, B). The same concordance between DNA biosynthesis and visual signal detection under day light conditions was found when neutral red was used as indicator (Fig. 2C).

**Fig. 3:**
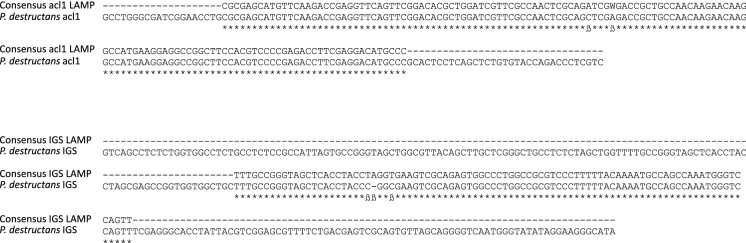
DNA-sequence based verification of the LAMP products obtained in the *acl1* (above) and *IGS* (below) based assays. Consensus sequences obtained from sequencing of the smallest DNA fragment in each assay were aligned to the respective reference sequences of target genes.-= nucleotide position not represented in consensus sequence, * = nucleotide position matching in consensus and reference sequences, ß = unmatched positions

Product verification was performed by comparing the nucleotide sequence of the smallest LAMP fragment with the respective template sequences. The partial sequences obtained for both LAMP products were 98.6% and 95.6% identical with the target sequences in *acl1* and *IGS*, respectively (Fig. 3). Although sequence identity was not 100%, as expected when using polymerases with low fidelity and comparing different isolates, results nevertheless suggest that both primers specifically amplify their target gene.

### Optimization of LAMP Protocols and Sensitivity Testing

To establish optimum incubation conditions for both LAMP assays, 12 LAMP reactions were prepared as previously described. Five microliters of purified genomic DNA from *P. destructans* OT-38-2010 were added as template. Reactions were incubated for 60 min in the heating block of a gradient thermal cycler, set to provide constant temperatures with 0.83 °C increments between 60.0 and 70.0 °C in individual wells. A clearly visible signal of bright green fluorescence occurred at temperatures between 62.8 and 65.5 °C for both primer sets. Incubation at lower and higher temperatures led to weak or no fluorescence, respectively. In subsequent experiments, *acl1*- and *IGS*-based reactions were incubated at 64.0 °C and 65.0 °C, respectively. Sensitivity of both assays was tested by setting up LAMP reactions using a tenfold serial dilution of OT-38-2010 genomic DNA as template. Reactions were incubated for 60 min. The *acl1*-based assay had a limit of detection (LOD) of 2.1 pg/rxn (Fig. S3 A). Based on a genome size of 35.818201 Mbp for *P. destructans* [51], this is equivalent to 54 theoretical genome copies per reaction. The same serial dilution of *P. destructans* OT-38-2010 DNA with the *IGS* specific primer set, using either calcein (Fig. S3 B) or neutral red (Fig. S3 C) as indicator, revealed an LOD of 21 fg/rxn for both indicators. This concentration was equivalent to 0.54 theoretical genomes per reaction [51]. Six repetitions of this experiment showed that the mentioned LOD was reached in about 50% of experiments suggesting an LOD of rather 1 genome equivalent per reaction. Table 3 gives an overview of the different LAMP experiments carried out in the laboratory as well as their detection limits.

**Table 3:** Overview over LAMP experiments made during the current study including different sampling techniques, sample preparation methods and primer sets with regard to the limit of detection (LOD)

Primer set	Sampling technique										
	LAMP with fungal material								LAMP with purified fungal DNA		
	Conidia per reaction cell disruption		PCM^a^ 3–17 dpi^b^		PCM 24–31 dpi		Soluble tape lifting	Field experiments with dry-LAMP (swab & tesafilm®	Calcein indicator	Neutral red indicator	V13-01184 indicator
	Without cell disruption	With cell disruption	Margin	Center	Margin	Center					
Pd-acl1-ID30	n.a.^c^	n.a	n.a	n.a	n.a	n.a	n.a	n.a	LOD^d^ 2.1 pg/reaction	LOD 2.1 pg/reaction	n.a
Pd-IGS-ID10	LOD 1.05 10^5^	LOD 1.05 10^3^	+^e^	+	+	−^e^	LOD 2.3 10^4^ con^f^/lifting	+	LOD 21 fg/reaction	LOD 21 fg/reaction	LOD 30 fg/reaction

^a^pure culture mycelium

^b^days post inoculation

^c^not applied

^d^Limit of detection

^e^ +  = LAMP signal detected, −  = no LAMP signal detected

^f^conidia

**Fig. 4:**
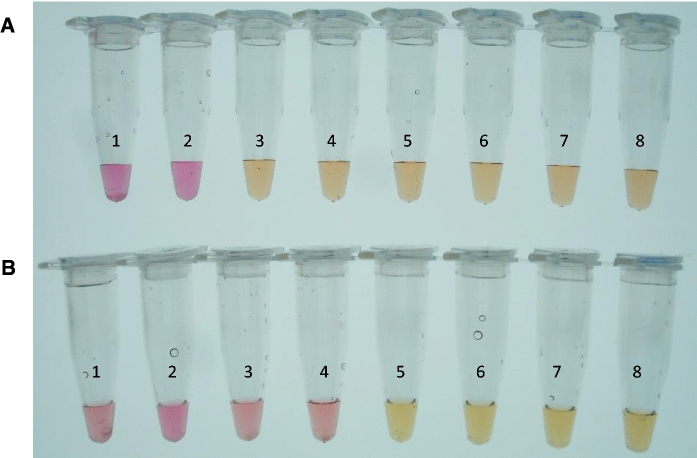
Sensitivity of the *IGS*-based LAMP assay for the detection of *P. destructans* conidia after direct addition to the master mix. Reactions were run under the conditions described in Table 2 with neutral red as indicator and a serial dilution of conidia from *P. destructans* OT-38-2010 as template. **A** LAMP assay with untreated conidial suspensions. **B** LAMP assay with conidial suspensions after vortexing with sterile glass beads. 1 = 1.05 × 10^6^ conidia /rxn; 2 = 1.05 × 10^5^ conidia/rxn; 3 = 1.05 × 10^4^ conidia/rxn; 4 = 1.05 × 10^3^ conidia/rxn; 5 = 1.05 × 10^2^ conidia/rxn; 6 = 1.05 × 10^1^ conidia/rxn; 7 = 1.05 conidia/rxn; 8 = 0 conidia/rxn

Sensitivity of the *IGS*-based LAMP assay for detection of *P. destructans* spores after direct addition to the master mix was tested with untreated conidia of isolate OT-38-2010. Five microliters per reaction were added directly to the master mix from a tenfold serial dilution of a stock solution containing 2.1 × 10^8^ conidia mL^−1^. In order to increase the sensitivity of direct spore analysis, 300 µl from each tenfold dilution was mixed with sterile glass beads and vortexed as described in materials and methods. Following the treatment, 5 µL from each dilution were transferred to a LAMP master mix and incubated at 65.0 °C for 60 min. The assay was sensitive to the detection of 1.05 × 10^5^ conidia per reaction without any treatment and 1.05 × 10^3^ conidia per reaction after simple glass beads vortex treatment with neutral red as indicator (Fig. 4). These results show that a simple mechanical treatment of conidia during sample preparation can increase the sensitivity of the assay by 100-fold.

### Specificity Testing of LAMP Assays

Both LAMP assays successfully amplified for the diverse set of 53 *P. destructans* isolates tested. No positive reactions were obtained with DNA from any other of the 159 fungal species tested, including the 25 *Pseudogymnoascus* species/variants herein tested (see Table S1 in supplemental materials). This result suggests that both LAMP assays are highly specific for the detection of *P. destructans* and that there will be no cross-reactions with the various taxonomic variants of *P. destructans* that are phylogenetically closely related and may co-occur in bat environments.

### Direct Testing of Pure Culture Mycelia

Signals were detected as early as 3 d post inoculation showing that the assay can be used on young cultures, obviating the need of waiting for the production of conidia. A signal was also detected, when material from older colonies was used. However, no signal was detected from colonies as soon as production of guttation droplets occurred indicating the presence of LAMP inhibitors. However, material taken from the droplet free margin of older colonies still gave a positive signal in the assay when colonies were at least 4 weeks old.

### Analysis of Tape Liftings

**Fig. 5:**
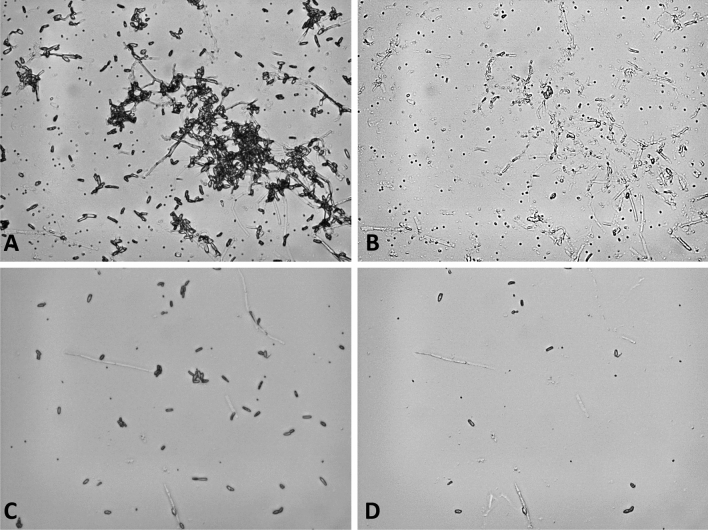
Tape lifting of *P. destructans* spores from a glass surface using 1″ Mask Plus II water soluble wave solder tape (spores per spot = number of spores dried on surface from 100 µL of spore suspension). **A** Suspension of 2.3 × 10^6^ spores per spot of *P. destructans* OT-38–2010 air-dried onto surface of a microscope glass slide, 400× magnification. **B** The same section of the surface after removal of spores with water soluble adhesive tape, 400× magnification. **C** Suspension of 2.3× 10^5^ spores per spot of *P. destructans* OT-38-2010 air-dried onto surface of a microscope glass slide, 400× magnification. **D** The same section of the surface after removal of spores with water soluble adhesive tape, 400× magnification

**Fig. 6:**
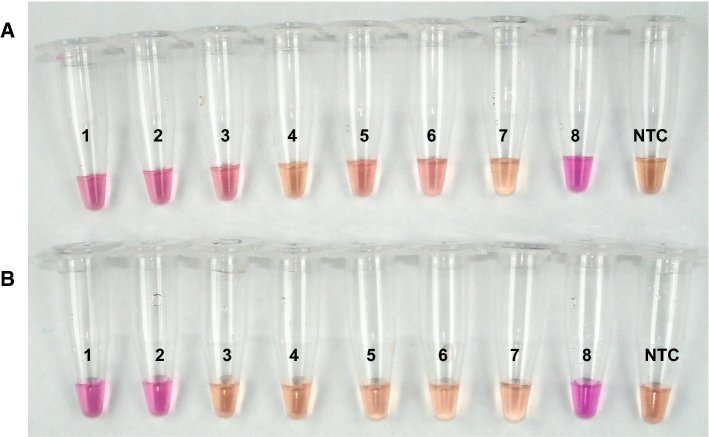
LAMP reaction with direct addition of: **A** fungal material prepared according to the new protocol from tape liftings of spores dried previously onto a glass surface, **B** Conidia added directly from a tenfold serial dilution of *P. destructans* OT-38-2010 conidial suspension. 1 = 2.3 × 10^6^ conidia per spot (**A**)/2.3 × 10^7^ conidia per mL (**B**); 2 = 2.3 × 10^5^ per spot/2.3 × 10^6^ per mL, 3 = 2.3 × 10^4^ conidia per spot/2.3 × 10^5^ per mL; 4 = 2.3 × 10^3^ per spot/2.3 × 10^4^ per mL; 5 = 2.3 × 10^2^ per spot/2.3 × 10^3^ per mL; 6 = 23 per spot/230 per mL, 7 = 0 per spot/0 per ml, 8 = positive control with 1.0 pg/rxn gDNA of *P. destructans* OT-38-2010, NTC = no template control with water instead of DNA. Pink color = positive reaction, yellow color = negative reaction

A protocol was developed in which water-soluble adhesive tape was used to lift fungal material from the surface of agar cultures and microscope glass slides as a model surface. First experiments showed that the tape material readily dissolved in demineralized water at 65.0 °C. However, using this method, a pellet consisting of globular microscopic structures remained after centrifugation and washing which inhibited the LAMP reaction (result not shown). We therefore tested different chaotropic compounds to enhance solubility of the material. Tests showed that 8 M urea resulted in complete disintegration of the tape material with no inhibition of the LAMP assays after three washings with demineralized water. Analysis of solution behaviour at different temperatures showed that the material dissolved completely even at ambient temperature of 23 °C. Figure 5A, B show the efficiency with which spores were lifted from a glass surface using the water soluble Mark Plus II tape. It was noteworthy that the tape removed spores with an efficiency close to 100% while leaving mycelial fragments on the surface. Lifting of spores from a tenfold serial dilution before preparation of spores for the LAMP assay using glass beads as described previously showed that a minimum of 2.3 10^4^ conidia per lifting were detected (see Fig. 6).

### Field Experiment: Testing Different Sample Types in Dry-LAMP Reactions

Four out of five samples from bats that have been visually classified as *P. destructans*-infected gave positive results in the dry-LAMP assay; the one ‘false’ negative was a sample that contained yellowish fluid (suspected inhibitor) which was collected from the bat together with the fungal material. One out of three samples from visually non-infected bats was positive in the LAMP assay. Two out of 11 swabs from hibernacula walls, where *P. destructans*-infected bats had been previously recorded, were positive in the LAMP assay. Two negative controls (one swab transferred in the tube within the hibernaculum and one during the LAMP preparation) were negative in the LAMP assay. Two tape liftings (tesafilm®) from *P. destructans*-infected bats were positive in the LAMP assay, the negative control (tape collected on a microscope slide without previously touching a bat) was negative. An overview of the results obtained with field samples is given in supplementary Table S2.

## Discussion

### Specificity of the LAMP Assays

We herein developed two LAMP assays for the detection of *P. destructans*. The *acl1*-based assay detects a typical single copy fungal housekeeping gene. The same gene was used by Niessen, Gräfenhan [52] as target sequence in a *Fusarium tricinctum* specific LAMP assay. Authors made use of the *acl1* sequence because it readily separated *F. tricinctum* from the closely related *F. avenaceum* which was not possible when other typical genes such as ß-tubulin, EF1α or calmodulin were targeted. The *IGS*-based assay developed here for *P. destructans* uses primers specific for a region that separates eukaryotic cistrons coding for different units of ribosomal RNA. The non-transcribed sequences usually display a high degree of variation between species since mutations are less subject to selection and can therefore be used to separate genetically closely related taxa such as the pathogenic *P. destructans* and the various close relatives that may occur in the same environments [6]. The specificity of our LAMP-assays was tested against gDNA of a wide variety of fungal species (Table S1), many of which (or their conspecifics) have been found in underground sites used by bats (e.g. [53–56]). The absence of cross-species reaction demonstrates the high specificity of our LAMP assays. However, the in silico analysis of primer binding revealed that the *IGS* primer set can be expected to have a superior selectivity for *P. destructans* since the number of mismatches with closely related taxa was much higher when compared with the *acl1* primer set. The higher the mismatch rate of a primer set with closely related non-target species, the higher the selectivity for the target species.

### Sensitivity of the LAMP Assays

As fungal cells usually contain several up to several hundred copies of rRNA coding genes [57], an assay based on one of those units, i.e. the *IGS* sequence, is supposed to provide a higher number of primer binding sites per DNA molecule and should thus have a higher sensitivity for the detection of its target organism. Our results fully confirmed this expectation because the *IGS*-based assay was about 100-fold more sensitive compared to the single-copy gene *acl1* assay. Depending on the detection system applied, the *IGS*-based LAMP assay had an LOD of 21–30 fg per reaction which is equivalent to 0.54–0.85 theoretical genome copies, respectively. Comparison to LOD of PCR or qPCR assays for *P. destructans* shows that the new assay has a similarly high sensitivity as the multicopy *ITS*- and *IGS*-based qPCR assays described previously [27, 28]. The *acl1*-based singlecopy LAMP assay had an LOD of 2.1 pg per reaction (equivalent to 54 genome copies per reaction) and was slightly less sensitive than the *alr*-gene based qPCR published by Chaturvedi, Rudd [26] with an LOD of 0.8 pg (equivalent to 20.7 genome copies per reaction). The *IGS*-based LAMP assay had a sufficiently high sensitivity to detect *P. destructans* even in samples with a low concentration of the pathogen. *IGS*-based LAMP assays were also developed for other fungi such as *Metharizium anisopliae* [58], *Fusarium oxysporum* f. sp. *cubense* tropical race 4 [59] or *Trichosporon asahii* and *T. mucoides* [60]. All these assays were also highly sensitive for their respective target organisms. Because of its higher sensitivity, the *IGS*-based LAMP assay should be given priority over the *acl1*-based assay when used in practical applications such as screening for the fungus in bat environments (e.g. hibernacula walls [20, 61, 62]).

### Advantages and Disadvantages of the LAMP Assay

In contrast to qPCR-based assays, LAMP is a useful tool for presence/absence assessment rather than for precise quantification of target organisms. Even though there is a correlation between time to result and the initial concentration of target DNA, the reaction runs with a dynamic that is too high and with moderate replicability to allow for precise DNA quantification. Few attempts were made in the past to apply LAMP in quantitative assays, but the results were generally less accurate as compared to qPCR [63, 64]. It was demonstrated that LAMP is rather insensitive to matrix inhibition in qualitative assays but shows a strong influence on the quantitative results [65].

Even though LAMP assays, similarly to other DNA- or RNA-based detection methods, do not provide direct information on the extent of wing damage or other symptoms nor information on the viability of their target organisms [66], the LAMP method presents several important advantages over qPCR. First, although extracted DNA can be used as template in the LAMP, as demonstrated herein, unextracted material can equally be used as template, providing a great advantage for applications in the field where limited laboratory equipment is available. Results presented herein show that a limit of 1.05 10^3^ conidia can be detected per reaction after minimal mechanical treatment from a solution of pure culture conidia. This LOD should be sufficiently sensitive to detect fungal colonization during early infection stages. Since *P. destructans* is a strongly sporulating species, even a detection limit of 1.05 10^5^ conidia per reaction that was obtained for pure culture conidia without mechanical treatment should be sufficient for the diagnosis of visible fungal infections. Second, a thermocycler is not needed to perform the LAMP as the amplification only requires a constant temperature, which can be obtained via a thermal block or even in a water bath (as demonstrated in our field trial, see below). Third, when using indicator dyes for colour change inside the reaction tube, the result is directly readable to the naked eye without the need for any further equipment. Therefore, these LAMP assays provide a greatly needed field-deployable molecular genetic tool for *P. destructans* detection that will considerably reduce the time necessary for accurate diagnosis. Given that the assays can directly use unextracted material as template, the LAMP assays also provide a quick and cheap method for genetically confirming species ID from culture material, adhesive tape samples or swab samples as classically used (e.g. [7–9, 11, 67]). These analyses can be performed in the field but also in the lab where they will provide considerable savings in terms of time and costs compared to qPCR. This new diagnostic LAMP assay and the recently published Visual-Pd scoring system [66] offer significant additions to the toolbox to better understand and monitor White-Nose Disease in bats. While the Visual-Pd score can be considered as non-invasive [66], the collection of samples from bats necessitates a soft contact between the collection item (swab, tape) and the animal skin. However, this can be achieved with minimal or even without disturbance when the procedure is carried out carefully and swiftly, in compliance with general arousal-avoidance measures during the hibernacula surveys [66].

### Swabbing or Tape Lifting in Combination with LAMP Assay as a Field-Deployable Method

The current study includes the development of a new method for the sampling of fungal material from solid surfaces and the preparation of such samples for application in LAMP assays. Results showed that spores were detected from a model glass surface with an LOD of 2.3 × 10^4^. Swabbing and tape lifting are common techniques in criminal forensics or veterinary medical mycology and are also used to sample bats for the presence of *P. destructans* [7, 22]. Tape lifting is particularly useful to obtain a rapid morphological identification of *P. destructans* by using light microscopy [7]. However, once the fungal material sticks to such tape, it appears difficult to recover it easily for further analyses. Thus, we tested both, common tesafilm® as well as tape lifting material which is water soluble, for direct amplification of the lifted material in the newly developed LAMP assay.

Our tests involved samples from pure cultures as well as swabs and tape liftings collected from bats and hibernacula walls. All sample types were rinsed directly in the LAMP buffer and an aliquot was directly used as LAMP target without further manipulation. Additionally, we developed and successfully tested a dried version of the LAMP reaction (‘dry-LAMP’) which is particularly convenient for shipment and storage prior to testing samples in the field. Although the sample sizes are low, bat swabs and tape samples were successfully tested under field conditions, providing a proof-of-concept for the applicability of the method in the field. Further investigations and optimization are nevertheless necessary before the dry-LAMP can be applied at large scale, especially for hibernacula wall swabs. Indeed, some negative results could be due to inhibition caused by impure sampling material [34] or might have been caused by the absence of high spore loads on the tested hibernacula walls.

The simplicity of swabbing or tape lifting and sample preparation, in combination with a rapid and sensitive LAMP assay as a field deployable detection tool, provides great practical potential to the newly developed methods.

Moreover, the sample preparation protocol is not restricted to applications involving LAMP but can also be used to prepare amplifiable DNA for PCR or qPCR applications. Specifically, the application of swabs or tape that can be tested in the field can be used much to the advantage of researchers, wildlife and conservation biologists involved in WND studies or even for the study of other dermatophytes in other animals or even in humans [35].

## Supplementary Information

Below is the link to the electronic supplementary material.

Supplementary file5 (DOCX 16 KB)

Figure S1 Binding site homologies between primer set Pd-acl1-ID30 and acl1 gene sequences of fungal species retrieved from GenBank. The used sequences shared >85 % total homology with the P. destructans acl1 gene sequence. Nucleotide positions with homology to the respective positions in the primer sequences are marked in grey, non-homologues positions are marked in white (PDF 16 KB)

Figure S2 Binding site homologies between primer set Pd-IGS-ID10 and the 28S-18S intergenic spacer (IGS) sequence of three P. destructans strains and 10 Pseudogymnoascus spp. and P. verrucosus. Sequences retrieved from GenBank. The used sequences shared >90 % total homology with the corresponding P. destructans sequence. Nucleotide positions with homology to the respective positions in the primer sequences marked in grey, non-homologues positions marked in white (PDF 531 KB)

Figure S3 Sensitivity of LAMP assays for the detection of P. destructans purified gDNA. Reactions were run under the conditions described in table 3 with calcein and neutral red as indicator dyes and a serial 10fold dilution of purified gDNA of strain OT-38-2010 as template. A LAMP assay using primer set Pd-acl1-ID30 with calcein indicator under UV light. Bright green fluorescence = positive reaction; no fluorescence = negative reaction. B LAMP assay using primer set Pd-IGS-ID10 with calcein indicator under UV light. C LAMP assay using primer set Pd-IGS-ID10 with neutral red indicator under day light conditions (pink = positive reaction; yellow = negative reaction). 1 = 213 ng per reaction (ng/rxn) of P. destructans OT-38-2010 gDNA; 2 = 21.3 ng/rxn; 3 = 2.1 ng/rxn; 4 = 210 pg/rxn; 5 = 21 pg/rxn; 6 = 2.1 pg/rxn; 7 = 210 fg/rxn; 8 = 21 fg/rxn; 9 = 2.1 fg/rxn; 10 = 210 ag/rxn; NTC = not template control, demineralized water instead of DNA-template (PDF 351 KB)

Supplementary file5 (XLSX 48 KB)

Supplementary file5 (DOCX 19 KB)

## Data Availability

All data have been published, either as part of the manuscript or as supplemental materials.

## Materials Availability

Fungal strains have been deposited in the culture collection of Chair of Microbiology at Technical University of Munich, Freising, Germany (all TMW clones) or at the culture collection of ISEM, University of Montpellier, France.
