# Supplementary material for: Development and Application of Loop-Mediated Isothermal Amplification (LAMP) Assays for Rapid Diagnosis of the Bat White-Nose Disease Fungus Pseudogymnoascus destructans
Source: Mycopathologia. 2022 Aug 5;187(5-6):547–65. doi: 10.1007/s11046-022-00650-9 (PMC9675650; doi:10.1007/s11046-022-00650-9)
Supplement: Supplementary file 1 — Supplementary file5 (DOCX 16 KB) [file 11046_2022_650_MOESM1_ESM.docx]

**Method description S1:**

**Sequencing of the acl1 gene in *P. destructans* OT-38-2010**

Presence and correctness of the acl1 sequence in the reference strain *P. de-structans* OT-38-2010 was checked by sequencing overlapping portions of the gene using primers Geo-acl1-f to Geo-acl1-r2 (see table 1). Sequencing templates were prepared in a conventional PCR set up with 2.5 µl 10x PCR buffer supplied together with the taq DNA polymerase (MPBiomedicals, Freiburg, Germany), 0.5 µl dNTP mix (10 mM each G, A, T, C], 0.5 µl each of forward and reverse primers (50 pmol/µl), 0.25 µl (5 U/µl) taq DNA polymerase, 1.0 µl of template DNA (ca. 50 ng/µl) and 19.75 µl deionized filter sterilized (0.2 µm membrane) water. PCR was run in a Mastercycler® gradient thermal cycler (Eppendorf, Hamburg, Germany) under the following conditions: 1 cycle of 4 min melting at 95 °C; 35 cycles of 1 min melting at 95 °C, 1 min annealing at 57 °C, 61.5 °C and 59 °C [primers Geo-acl1-f/, Geo-acl1-f1/r1, Geo-acl1-f2/r2, respectively, see table 1 for sequences], 60 s elongation at 72 °C; 1 cycle of 5 min at 72 °C for final elongation. PCR reactions were separated on 1.3 % agarose gels (LE agarose, Biozym, Hessisch Olendorf, Germany) prepared with TAE buffer at 100 V, 95 mAmp. Following separation, gels were stained in dimidium bromide (Carl Roth, Karlsruhe, Germany) and inspected on a UV transilluminator model 28M (Herolab, Wiesloch, Germany). Gels were documented using a video documentation system (Intas, Göt-tingen, Germany). For sequencing, bands of interest were cut out of the gel and DNA was isolated using the peqGold gel extraction kit (Peqlab, Erlangen, Germany). Bi-directional sequencing of the purified PCR products was performed by GATC (Freiburg, Germany) with the same primers used for their synthesis, respectively. Sequences were aligned to the acl1 sequence of *P. destructans* 20631-21 (Genbank accession number NW_020167528.1) using the BioEdit software package version 7.0.9.0 [(1)].

**Method description S2:**

**Preparation of tape lifting samples using water soluble tape material**

The water soluble tape (1” Mask Plus II, 3M, Maplewood, MS, USA) was solubilized by addition of 2 ml sterile 8 M aqueous urea and incubated at ambient temperature (23 °C) for 5 min with hand mixing of the sample every minute. The sample was centrifuged for 2 min at 18,500 x g at 23 °C and the supernatant discarded subsequently. The pellet was dissolved in 1 ml 8 M aqueous urea and incubated for another 5 min under the conditions described previously. The supernatant was discarded and the pellet washed 3x with 1 ml of sterile demineralized water by mixing on a vortex or flicking the tube for homogenization and subsequent centrifugation for 2 min at 18,500 x g (23 °C). After discarding the supernatant of the final washing, the pellet was suspended in 100 µl of sterile demineralized water. Five microliters of the homogenized sample were used directly as LAMP template. To obtain a higher sensitivity, the pellet was alternatively dissolved in 300 µl of sterile deionized water and the complete volume transferred to a sterile 1.5 ml reaction tube filled with a mixture of sterile glass beads (0.4 g of 0.5 mm diameter, 0.22 g of 1.25–1.65 mm diameter) and vortexed for 6 min at maximum frequency at ambient temperature. Again, 5 µL of the solution were used as template in the LAMP assay.

1. Busam G, Kassemeyer H-H, Matern U. Differential expression of chintinases in *Vitis vinifera* L. responding to systemic acquired resistance activators or fungal challenge. Plant Physiol. 1997;115:1029-38.
