# Supplementary material for: Development and Application of Loop-Mediated Isothermal Amplification (LAMP) Assays for Rapid Diagnosis of the Bat White-Nose Disease Fungus Pseudogymnoascus destructans
Source: Mycopathologia. 2022 Aug 5;187(5-6):547–65. doi: 10.1007/s11046-022-00650-9 (PMC9675650; doi:10.1007/s11046-022-00650-9)
Supplement: Supplementary file 2 — Figure S1 Binding site homologies between primer set Pd-acl1-ID30 and acl1 gene sequences of fungal species retrieved from GenBank. The used sequences shared >85 % total homology with the P. destructans acl1 gene sequence. Nucleotide positions with homology to the respective positions in the primer sequences are marked in grey, non-homologues positions are marked in white (PDF 16 KB) [file 11046_2022_650_MOESM2_ESM.pdf]

Figure S1

Primer acl1-ID30	F3	F2	LF rc[1]	F1c rc
Sequence 5´-> 3´	**ATTGTTGCCTGGGCGATC**	**CGCGAGCATGTTCAAGACC**	**AGGTTCAGTTCGGACACGCT**	**GGATCGTTCGCCAACTCGCAG**
Ps.[2] destructans OT38-2010 (JN242242.1)[7]	ATTGTTGCCTGGGCGATC	CGCGAGCATGTTCAAGACC	AGGTTCAGTTCGGACACGCT	GGATCGTTCGCCAACTCGCAG
Ps. destructans 20631-21 (XM_024468044.1)	ATTGTTGCCTGGGCGATC	CGCGAGCATGTTCAAGACC	AGGTTCAGTTCGGACACGCT	GGATCGTTCGCCAACTCGCAG
Ps. verrucosus UAMH 10579 (KV460245.1)	ATTGTTGCCTGGGCGATC	CGCGAGCATGTTCAAGACC	AGGTTCAGTTCGGACACGCT	GGATCGTTCGCCAACTCGCAG
Ps. pannorum BBA 66108 (JN571714.1)	ATTGTTGCCTGGGCCATT	CGCGAGCATGTTCAAGACC	AAGTTCAGTTCGGACACGCT	GGATCGTTCGCCAACTCGCAG
V.[3] dahliae VdLs.17 (CP010984.1)	ATTGTTGCCTGGGCCATT	CGCCAGCATGTTCAAGACT	AGGTTCAGTTCGGCCACGCC	GGTGCCTTTGCCAACTCAACG
Pod.[4] anserina S mat+ (CU638743.1)	ATTGTCGCGTGGGCTATC	CGCCAGCATGTTCAAGACT	AGGTTCAGTTCGGCCACGCT	GGTGCCTTTGCCAATTCTCAG
Pod. comata T (LR026966.1)	ATTGTCGCGTGGGCTATC	CGCCAGCATGTTCAAAACT	AGGTTCAGTTCGGCCACGCT	GGTGCCTTTGCCAATTCTCAG
Pen.[5] chrysogenum Wisconsin 54-1255 (XM_002568989.1)	GTCGTCGCCTGGGCTATC	CGCCAGCATGTTCAAGACC	AGGTCCAGTTCGGTCACGCC	GGTGCCTCCGCCAACTCCGAT
Beau.[6] bassiana ARSEF 2860 (XM_008603705.1)	ATTGTCGCATGGGCCATT	TGCCAGCATGTTCAAGACC	AGGTCCAATTCGGTCACGCT	GGTGCCCTTGCCAACTCTCAG

Primer acl1-ID30	B3 rc	B2 rc	LB	B1c
Sequence 5´-> 3´	**GCACTCCTCAGCTCTGTGTA**	**ACCTTCGAGGACATGCCC**	**TGAAGGAGGCCGGCTTCCA**	**GACCGCTGCCAACAAGAACAAG**
Ps. destructans OT38-2010 (JN242242.1)	GCACTCCTCAGCTCTGTGTA	ACCTTCGAGGACATGCCC	TGAAGGAGGCCGGCTTCCA	GACCGCTGCCAACAAGAACAAG
Ps. destructans 20631-21 (XM_024468044.1)	GCACTCCTCAGCTCTGTGTA	ACCTTCGAGGACATGCCC	TGAAGGAGGCCGGCTTCCA	GACCGCTGCCAACAAGAACAAG
Ps. verrucosus UAMH 10579 (KV460245.1)	GCTCTCCTCAGCCAGGTTTA	ACCTTCGAGGATATGCCC	TGAAGGAGGCCGGCTTCCA	GACCGCTGCCAACAAGAACAAG
Ps. pannorum BBA 66108 (JN571714.1)	GCTCTTCTCAGCTCCGTTTA	ACCTTCGAGGACATGCCC	TGAAGGAGGCCGGATTCCA	GACCGCTGCCAACAAGAACAAG
V. dahliae VdLs.17 (CP010984.1)	AACGTCCTCAAGCAGGTCTA	ACCTTTGAGGACATGCCC	TGAAGGAGGCCGGCTTCCA	GACTGCCAAGACCAAGAACGAG
Pod. anserina S mat+ (CU638743.1)	GCTCTCCTCGGGCAGGTGTA	ACCTTCGAGGACATGCCC	TGAGGGAGGCCGGTTTCTA	AACTGCCAAGATGAAGAACGAG
Pod. comata T (LR026966.1)	GCTCTCCTCAGGCAGGTGTA	ACCTTCGAGGACATGCCC	TGAGGGAGGCCGGTTTCTA	AACTGCCAAGATGAAGAACGAG
Pen. chrysogenum Wisconsin 54-1255 (XM_002568989.1)	CAGATGCTCAAGCAGGTTTA	ACCTTCGAGGATCTCCCC	TGCGCGAAGCTGGCATCCA	GACTGCCGTCACCAAGAACAAG
Beauveria bassiana ARSEF 2860 (XM_008603705.1)	AGTGTTCTTGCTGAGCTGTA	ACTTTTGAGGACATGCCC	TGAAGGAGGCCGGCTTCCA	GACCGCTGCCACCAAGAACAAG

[1]reverse complement of original primer sequence

[2]Pseudogymnoascus

[3]Verticillium

[4]Podospora

[5]Penicillium

[6]Beauveria

[7]GenBank accession number
