# Supplementary material for: Development and Application of Loop-Mediated Isothermal Amplification (LAMP) Assays for Rapid Diagnosis of the Bat White-Nose Disease Fungus Pseudogymnoascus destructans
Source: Mycopathologia. 2022 Aug 5;187(5-6):547–65. doi: 10.1007/s11046-022-00650-9 (PMC9675650; doi:10.1007/s11046-022-00650-9)
Supplement: Supplementary file 3 — Figure S2 Binding site homologies between primer set Pd-IGS-ID10 and the 28S-18S intergenic spacer (IGS) sequence of three P. destructans strains and 10 Pseudogymnoascus spp. and P. verrucosus. Sequences retrieved from GenBank. The used sequences shared >90 % total homology with the corresponding P. destructans sequence. Nucleotide positions with homology to the respective positions in the primer sequences marked in grey, non-homologues positions marked in white (PDF 531 KB) [file 11046_2022_650_MOESM3_ESM.pdf]

Figure S2

Primer set IGS-ID10	F3	F2	LF2 rc[1]	LF1 rc (alternative)	F1c rc
Sequence 5´-> 3´	**CCTCTCCGCCATTAGTGC**	**CTGGCGTTACAGCTTGCT**	**AGCCGGTGGTGGCTGCTTT**	**GCTGCCTCTCTAGCTGGTT**	**GCCGGGTAGCTCACCTACCT**
Ps.[2] destructans 01NH07 (JX270192.1)[3]	CCTCTCCGCCATTAGTGC	CTGGCGTTACAGCTTGCT	AGCCGGTGGTGGCTGCTTT	GCTGCCTCTCTAGCTGGTT	GCCGGGTAGCTCACCTACCT
Ps. destructans 20631-21 (JX415267.1)	CCTCTCCGCCATTAGTGC	CTGGCGTTACAGCTTGCT	AGCCGGTGGTGGCTGCTTT	GCTGCCTCTCTAGCTGGTT	GCCGGGTAGCTCACCTACCT
Ps. spec. 24MN13 (JX270328.1)	CCTAGGGCTCCACGGGGT	CGTCCCCTGGAACGGTCC	CTGGCTTTGCCGGGTAGCT	ATGGGGCCTTTGCCTCTCC	GCCGGGTAGCTAGCCTCACA
Ps. spec. 20KY12 (JX270296.1)	CCGGGTGGCTTTGGCATA	AGGGTCCTACAGAGAGTT	TCCCTACAGGGCATTTGCC	ATGGGGGTCTTCTCCCCTA	GCCGGGTAGCTAGCCTCACA
Ps. spec. 17WV04 (JX270270.1)	CCGGGTGGCTTTGGCATA	AGGGTCCTACAGAGAGTT	TCCCTACAGGGCATTTGCC	ATGGGGGTCTTCTCCCCTA	GCCGGGTAGCTAGCCTCACA
Ps. spec. 14PA06 (JX270249.1)	CCGGGTGGCTTTGGCATA	AGGGTCCTACAGAGAGTT	TCCCTACAGGGCATTTGCC	ATGGGGGTCTTCTCCCCTA	GCCGGGTAGCTATAGCCCTA
Ps. spec. 24MN11 (JX270327.1)	CCCGTGTGGCTTTGGGGT	AGGGTTCTACAGGGTTCC	TCCCTACAGGGCCGTTCCT	TTAGGGGCCTTGTCCCCTA	GCCGGGTAGCTATAGCCCTA
Ps. spec. 15PA10B (JX270257.1	CCCGTGTGGCTTTGGGGT	AGGGTTCTACAGGGTTTC	TCCCTACAGGGCCGTTCCT	TTAGGGGCCTTGTCCCCTA	GCCGGGTAGCTATAGCTCTA
Ps. spec. 21IN05 (JX270300.1)	CCCGTGTGGCTTTGGGGT	AGGGTTCTACAGGGTTCC	TCCCTACAGGGCCGTTCCT	TTAGGGGCCTTGTCCCCTA	GCCGGGCAGCTATAGCCCTA
Ps. spec. 11MA07 (JX270236.1	CCCGTGTGGCTTTGGGGT	AGGGTACTACAGGGTTCC	TCCCTACAGGGCATTTGCC	TTGGGGGCCTTGCCCCCTA	ATACCAAGGGTCCCCACCTA
Ps. spec. 07MA14 (JX270222.1)	GCTGCAGGGCTTCACGGG	CCTAGCTTTGGGGCTGTT	TCCCCACCTAGCATTTGCC	AGGAGAGCTCTGGGGTGTC	GCACGGGGGGTGTTGTGCCC
Ps. spec. 18VA16 (JX270281.1)	CCTACAGCTCTGCAGGGC	CGGGGCCTCGGGCCTGTT	GGCTCGTGGTGCTGCGAGC	GGGGACCTCCAGGGTGTAG	GCCGGGTAGCTCACCTACCT
Ps. verrucosus UAHM 10579 (NW_017263654.1)	CCTCTTCGCTATTATTGC	CCGGGTTACAGCTTCCTT	AGCCCGTGGTTACTGCTTT	AGTGCCTGTATGGCTGGTT	GCCGGGTAGTTCAGCTGCCC

Primer set IGS-ID10	B3 rc	B2 rc	LB	B1c
Sequence 5´-> 3´	**GCACCTATTACGTCGGAGC**	**CCAAATGGGTCCAGTTTCGA**	**CGCGTCCCTTTTTACAAAATGC**	**GAAGTCGCAGAGTGGCCCTG**
Ps. destructans 01NH07 (JX270192.1)	GCACCTATTACGTCGGAGC	CCAAATGGGTCCAGTTTCGA	CGCGTCCCTTTTTACAAAATGC	GAAGTCGCAGAGTGGCCCTG
Ps. destructans 20631-21 (JX415267.1	GCACCTATTACGTCGGAGC	CCAAATGGGTCCAGTTTCGA	CGCGTCCCTTTTTACAAAATGC	GAAGTCGCAGAGTGGCCCTG
Ps. spec. 24MN13 (JX270328.1)	GCCCACTGTGGTTGCTGAT	CCGGGTAGCTCACCTCCCGA	CGCGTCCCTTTTTACAAAACGC	CACCTCCCGAGCCTGCCTAC
Ps. spec. 20KY12 (JX270296.1)	GGGAGCCTGTGGTGGCCGC	CCGGGTAGCTCACCTACCCA	TACGTCCCTTTTTACAAAAACG	AGCCTCACAGATTGCAAGAG
Ps. spec. 17WV04 (JX270270.1)	GGGAGCCTGTGGTGGCCGC	CCGGGTAGCTCACCTACCCA	TACGTCCCTTTTTACAAAAACG	AGCCTCACAGATTGCAAGAG
Ps. spec. 14PA06 (JX270249.1)	GGGAGCCTGTGGTGGCCGC	CCGGGTAGCTCACCTACCCA	TACGTCCCTTTTTACAAAACGC	AGCCTCACAGATTGCAAGAG
Ps. spec. 24MN11 (JX270327.1)	GCAAGTCCGTGGTGGCCGC	CCGGGTAGCTCACCTACCCG	CGGGTAGCCCACCTACCCGGCA	TAGCCCTACAGATTCCGCCT
Ps. spec. 15PA10B (JX270257.1	GCAAGTCCGTGGTGGCCGC	CCGGGTAGCTCACCTACCCG	TACGTCTCTTTTTACAAAACGC	TAGCCCTACAGATTCCGCCT
Ps. spec. 21IN05 (JX270300.1)	GCAAGTCCGTGGTGGCCGC	CCGGGTAGCTCACCTACCCG	TACGTCTCTTTTTACAAAACGC	TAGCTCTACAGATTGCCAGA
Ps. spec. 11MA07 (JX270236.1	GCAAAACTGTAGTGTGGCC	CCGGGTAGCCCACCTACCCG	AGGGCACTTATACGTCGGAGCG	TAGCCCTACAGATTGCCTGA
Ps. spec. 07MA14 (JX270222.1)	GCTGCTGCTTTCTTCGCCT	CCGGGTAGCTGGTGTCTAGA	CCCGTCCCTTTTTTGAAAACAC	CCCCACCTAGCATTTGCCTC
Ps. spec. 18VA16 (JX270281.1)	GCAAGCCTGTGGTGGCTGT	CCGGGTAGCCCACCTACCCG	TACGTCTCTTTTTAAAAAAACA	TTGTGCCCTAGATGGCGCCT
Ps. verrucosus UAHM 10579 (NW_017263654.1)	GCTATTATTGCCGGGTTAC	CTCTTTGGGCGCAATTTTGA	CGAGTGTTTTCTGACGAGTCGC	AAAATCGCAGTTTGGCCCTA

[1]reverse complement of original primer sequence

[2]*Pseudogymnoascus*

[3]GenBank accession number
