# Supplementary figures and images for: Development and Application of Loop-Mediated Isothermal Amplification (LAMP) Assays for Rapid Diagnosis of the Bat White-Nose Disease Fungus Pseudogymnoascus destructans
Source: Mycopathologia. 2022 Aug 5;187(5-6):547–65. doi: 10.1007/s11046-022-00650-9 (PMC9675650; doi:10.1007/s11046-022-00650-9)

Figure S3

A

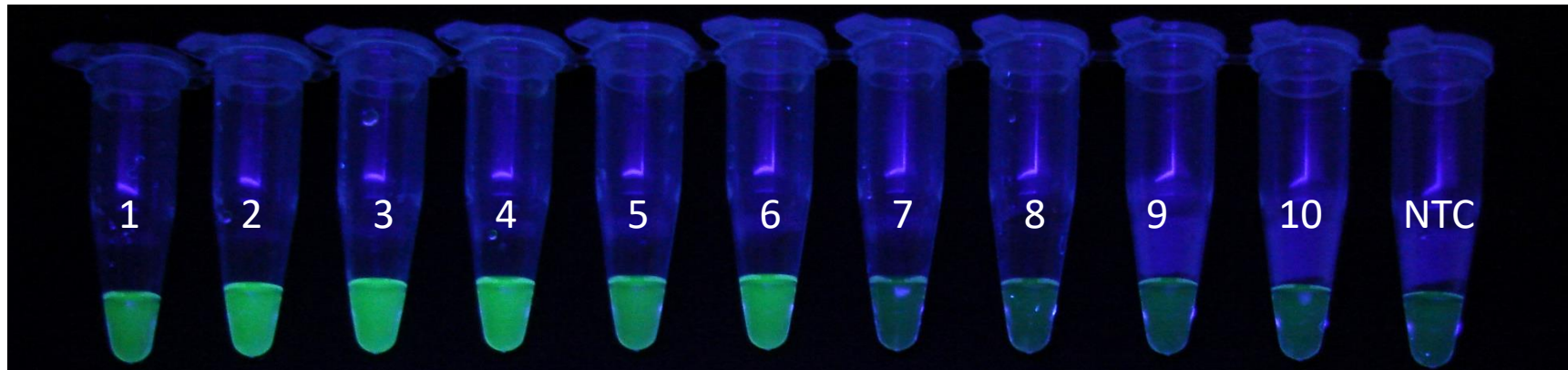

B

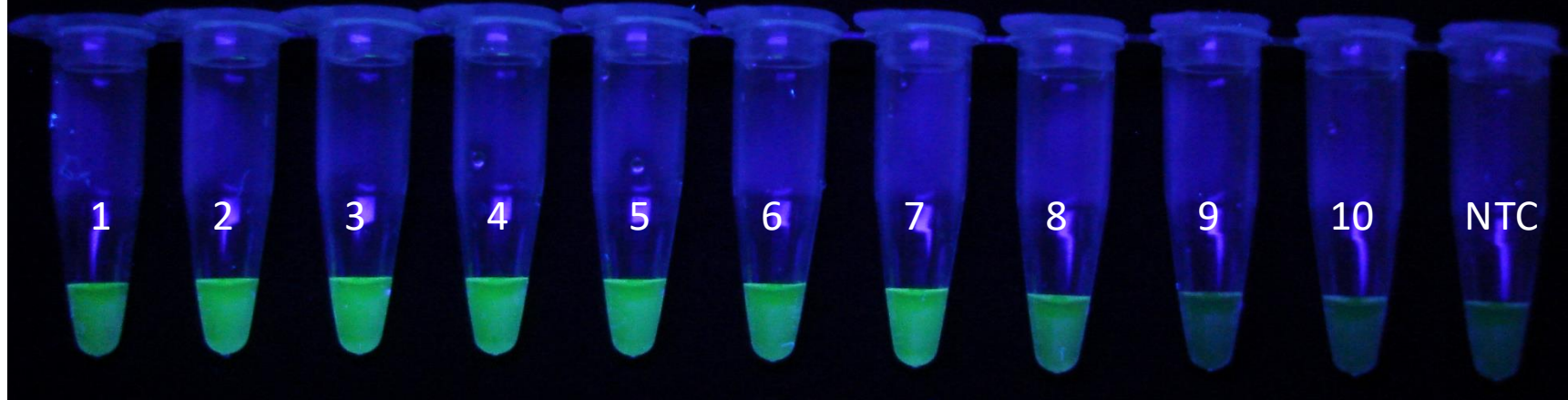

C

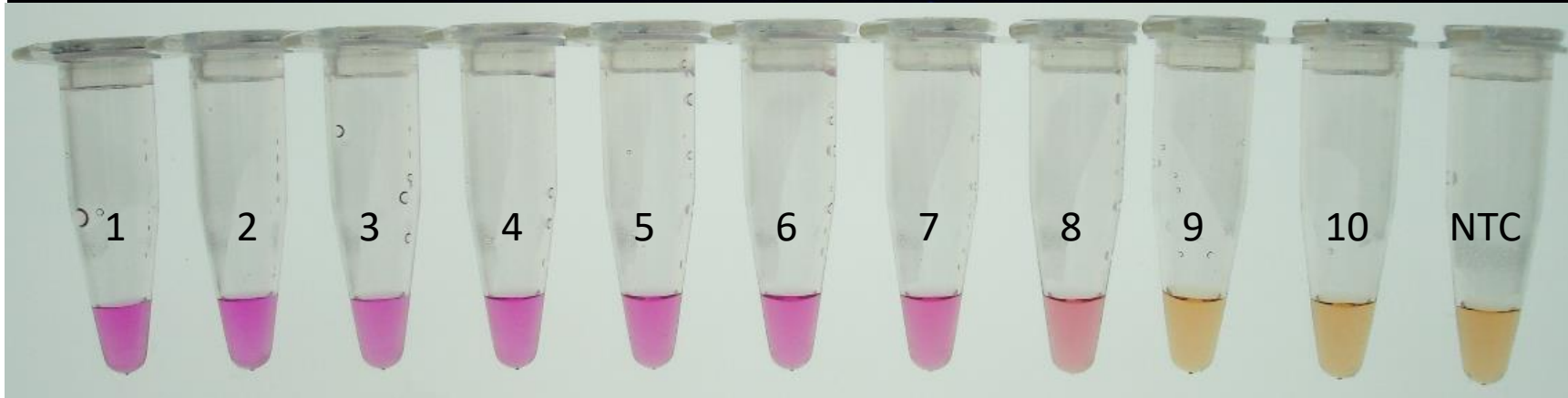

Supplement: Supplementary file 4 — Figure S3 Sensitivity of LAMP assays for the detection of P. destructans purified gDNA. Reactions were run under the conditions described in table 3 with calcein and neutral red as indicator dyes and a serial 10fold dilution of purified gDNA of strain OT-38-2010 as template. A LAMP assay using primer set Pd-acl1-ID30 with calcein indicator under UV light. Bright green fluorescence = positive reaction; no fluorescence = negative reaction. B LAMP assay using primer set Pd-IGS-ID10 with calcein indicator under UV light. C LAMP assay using primer set Pd-IGS-ID10 with neutral red indicator under day light conditions (pink = positive reaction; yellow = negative reaction). 1 = 213 ng per reaction (ng/rxn) of P. destructans OT-38-2010 gDNA; 2 = 21.3 ng/rxn; 3 = 2.1 ng/rxn; 4 = 210 pg/rxn; 5 = 21 pg/rxn; 6 = 2.1 pg/rxn; 7 = 210 fg/rxn; 8 = 21 fg/rxn; 9 = 2.1 fg/rxn; 10 = 210 ag/rxn; NTC = not template control, demineralized water instead of DNA-template (PDF 351 KB) [file 11046_2022_650_MOESM4_ESM.pdf]
