# Supplementary material for: Development and Application of Loop-Mediated Isothermal Amplification (LAMP) Assays for Rapid Diagnosis of the Bat White-Nose Disease Fungus Pseudogymnoascus destructans
Source: Mycopathologia. 2022 Aug 5;187(5-6):547–65. doi: 10.1007/s11046-022-00650-9 (PMC9675650; doi:10.1007/s11046-022-00650-9)
Supplement: Supplementary file 6 — Supplementary file5 (DOCX 19 KB) [file 11046_2022_650_MOESM6_ESM.docx]

**Table S2**: Results of the LAMP Field experiment. Different sampling types (swabs, tesa® tape liftings) and surfaces (bats, hibernacula walls) have been tested with dry-LAMP reactions. For sampling from bats, expected LAMP results are based on the visual Pd-score (a visual Pd-score=0 indicates no or cryptic *P. destructans*-infection while a visual Pd-score>0 indicates a *P. destructans*-infection; see Fritze et al. 2021)(1). Likely reasons for mismatches between expected and obtained LAMP results are explained in the comment column.

| **Sample ID** | **Sample type** | **Sample description** | **Expected LAMP result** | **true LAMP result** | **comment** |
| --- | --- | --- | --- | --- | --- |
| 12 | Bat swab | nose swab from a bat with Pd-score>0 | + | + |  |
| 14 | Bat Swab | nose swab from a bat with Pd-score>0 | + | - | probably inhibition due to yellow fluid collected with the sample (see (2, 3)) |
| 18 | Bat Swab | forearm swab from a bat with Pd-score>0 | + | + |  |
| 25 | Bat Swab | nose swab from a bat with Pd-score>0 | + | + |  |
| 39 | Bat swab | nose swab from a bat with Pd-score>0 | + | + |  |
| 26 | Bat Swab | nose swab from a bat with Pd-score=0 |  | + | probably cryptic infection (see (1)) |
| 28 | Bat Swab | forearm swab from a bat with Pd-score=0 |  | - |  |
| 29 | Bat Swab | forearm swab from a bat with Pd-score=0 |  | - |  |
| Neg-field1 | swab | negative control swab from the field | - | - |  |
| Neg-lab | swab | Negative control swab during reaction procedure | - | - |  |
| 40 | Wall swab | wall swab next to a bat; bats with Pd-score>0 have been observed at this position one month earlier |  | + |  |
| 42 | Wall swab | wall swab from a position where bats with Pd-score>0 have been observed one month earlier |  | - |  |
| 45 | Wall swab | wall swab from a position where bats with Pd-score>0 have been observed one month earlier |  | - |  |
| 47 | Wall swab | wall swab from a position where bats with Pd-score>0 have been observed one month earlier |  | - |  |
| 48 | Wall swab | wall swab next to a bat with Pd-score>0 |  | - |  |
| 30 | Wall Swab | wall swab next to a bat with Pd-score>0 |  | - |  |
| 31 | Wall Swab | wall swab next to a bat with Pd-score>0 |  | - |  |
| 41 | Wall swab | wall swab without positive bat nearby but in proximity of previously WND positive bats |  | - |  |
| 43 | Wall swab | wall swab from a position where bats with Pd-score>0 have been observed one month earlier |  | + |  |
| 44 | Wall swab | wall swab next to a bat with Pd-score>0 (also observed one month earlier). |  | - |  |
| 46 | Wall swab | wall swab without bats nearby |  | - |  |
| 32 | tesa tape | tape lifting from a bat with Pd-score>0 | + | + |  |
| 33 | tesa tape | tape lifting from a bat with Pd-score>0 | + | + |  |
| Neg-tape | tesa tape | Negative control tape | - | - |  |

1. Fritze M, Puechmaille SJ, Fickel J, Czirják GÁ, Voigt CC. A rapid, in-situ minimally-invasive technique to assess infections with *Pseudogymnoascus destructans* in bats. Acta Chiropterol. 2021;23:259-70.

2. Niessen L. Current state and future perspectives of loop-mediated isothermal amplification (LAMP)-based diagnosis of filamentous fungi and yeasts. Appl Microbiol Biotechnol. 2015;99(2):553-74.

3. Francois P, Tangomo M, Hibbs J, Bonetti E-J, Boehme CC, Notomi T, et al. Robustness of a loop-mediated isothermal amplification reaction for diagnostic applications. FEMS Immunol Med Microbiol. 2011;62(1):41-8.
